# WAVE~Ripples for Change Obesity Two-Year Intervention in High School Soccer Players: Process Evaluation, Best Practices, and Youth Engagement

**DOI:** 10.3390/nu10060711

**Published:** 2018-06-01

**Authors:** Yu Meng, Siew Sun Wong, Melinda M. Manore, Mēgan Patton-López

**Affiliations:** 1 School of Biological and Population Health Sciences, College of Public Health and Human Sciences, Oregon State University, Corvallis, OR 98331, USA; melinda.manore@oregonstate.edu; 2 Family and Community Health, School of Biological and Population Health Sciences, College of Public Health and Human Sciences Oregon State University, Corvallis, OR 98331, USA; siewsun.wong@oregonstate.edu; 3 Division of Health and Exercise Science, Western Oregon University, 240 Richard Woodcock Education Center, 345 Monmouth Ave N., Monmouth, OR 97361, USA; pattonlm@wou.edu

**Keywords:** process evaluation, fidelity, feasibility, sports nutrition, intervention, coach, sport team

## Abstract

This paper reports the process data on program fidelity, best practices for intervention implementation, youth and coach engagement, and youth application of knowledge and skills for the two-year WAVE~Ripples for Change (WAVE) obesity prevention intervention program focused on healthy eating, physical activity, and life skills with high school (HS) soccer players aged 14–19 years. Internal (staff: *n* = 7; volunteers: *n* = 27) and external (youth: *n* = 100; coaches: *n* = 9) stakeholders were interviewed/ surveyed. Staff rated program fidelity as high (94%), as did volunteers (85%). Best practices included coach encouragement for athlete participation, use of on-line consent for enrollment, building relationships with HS staff to complete assessments, sending text reminders, and providing incentives. Study results showed an enrollment rate of 72%, completion of baseline assessments of 89–98%, attendance of sports nutrition lessons in Year 1 and Year 2 of 90% and 39%, respectively, and team-building workshop (TBW) attendance of 25–31%. Activities exceeding youth expectations (>90%) included, (1) activities with their soccer team; (2) the TBW-cooking; and (3) sports nutrition lessons. The obesity prevention skills most applied by youth were obtained from the TBW-gardening and harvesting (49%), the TBW-cooking (43%), and sports nutrition lessons (44%). Coaches also rated the sports nutrition lessons highly and reported increased awareness for hydration/fueling during sport by the athletes. Using sport teams/clubs to engage youth in obesity prevention is a feasible model for future study.

## 1. Introduction

Childhood obesity is significantly more prevalent in adolescents aged 12–19 years old (20.6%) than in younger children (13.9–18.5%) [1]. One proposed childhood obesity prevention strategy is to encourage youth to participate in organized sports [2]. Currently, over 44 million North American youths participate in sports, with 54% of high school (HS) youth having reported participating in at least one sport [3]. However, sport participation does not mean these youth are not vulnerable to obesity. Among adolescents (aged 12–19 years) participating in leisure time sports, 16.6% of males and 15.3% of females were classified as obese (body mass index (BMI, kg/m^2^) >95th percentile for age and sex) [4]. Nelson et al. found that while youth who participated in sports ate more fruits, vegetables, and milk, they also ate more fast-food and sugar-sweetened beverages [5]. These latter two food groups increase the risk of obesity, especially if consumption continues once sports participation concludes and physical activity (PA) levels decline [6]. While youth participating in sports are more active than non-participants, Mäkelä et al. found that only 24% of sport participants obtained more than 60 min of moderate-to-vigorous PA (MVPA) per day [7]. Thus, many youth athletes may not be meeting the daily PA recommendation [8]. In general, research with HS athletes has focused primarily on preventing substance abuse and eating disorders, especially in female athletes [9,10,11]. Only one study, using a single male rugby player, has implemented a dietary intervention to improve nutrition knowledge and dietary quality, and promote positive body image among HS athletes [12]. No studies report promoting PA among HS athletes when they are not engaged in sports. To date, youths participating in sports have been overlooked by most researchers as a target audience for obesity prevention [13].

Soccer is one of the top five most popular HS sports for both males and females in the U.S., and is especially popular among Latinos [4]. Although Latina girls have lower sport participation rates in general, they are more likely to play soccer [4]. Manore et al. reported that Latino soccer players have lower sports nutrition knowledge scores (23% lower) and greater daily added sugar intake (12 g/d more) than non-Latino players [14,15], which can place them at risk of developing obesity in the future, especially if they no longer engage in sports. Thus, implementing obesity prevention with HS soccer players is a unique opportunity to engage with youth, improve their nutritional knowledge, and promote healthy diets and continued participation in PA. It is also an opportunity to engage under recruited groups, especially youth from ethnic minorities and various income levels, since soccer is such a universally popular sport [16].

The WAVE obesity prevention program included a two-year Physical Activity, Nutrition, and Family and Consumer Sciences (PAN-FCS) life skill-building intervention with HS soccer players (aged 14–19 years) [14,15,17]. The primary objective of the intervention was to promote healthy eating through sports nutrition education, maintenance of PA outside the soccer season, and life skills (e.g., meal planning, shopping, cooking, and gardening) for obesity prevention. A secondary objective was to test the feasibility of using a mixed virtual- and physical-world learning environment to reinforce sports nutrition knowledge for better sport performance, maintenance of PA, and adoption of health behavior changes for life-long obesity prevention. 

We assessed dietary intake (fruits, vegetables, saturated fat and added sugar) and PA (steps/day and exercise intensity) over the two-year intervention. Results showed that intervention participants significantly decreased added sugar (−12 g/day) and saturated fat (−2.7 g/day) intake, with no changes in fruit and vegetable intake. When differences between intervention and comparison groups were assessed, only added sugar was significantly lower in the intervention group (−10.4 g/day). This intervention group’s lower sugar intake was attributed to a decreased consumption of cake/cookie snacks, ice cream, and ice-cream bars. Results from PA assessments show that PA activity was significantly higher (9937 steps/day) during the soccer season as compared the off-season (8032 steps/day) [15]. Assessment of key quantitative outcomes alone does not provide a global understanding of program impacts, best practices, and intervention effectiveness with active youth.

To evaluate other aspects of the intervention, we collected process data on program fidelity, best practices for intervention implementation, youth and coach engagement in intervention activities, and youth application of knowledge and skills to their lives.

## 2. Materials and Methods

### 2.1. WAVE Program Backgroud

The WAVE~Ripples for Change (WAVE) program is a two-year integrated (research, education and extension) obesity prevention intervention targeting HS soccer players (aged 14–19 years). The intervention was age-specific and included face-to-face sports nutrition lessons, PA assessments, and team-building workshops (TBWs), and used experiential learning in a virtual world (VW) environment to reinforce these lessons. The VW learning occurred in Rippleville VW (the WAVE Study VW grid) within the OpenSimulator platform, which is an open source version of Second Life. The WAVE educational objectives were to teach PAN-FCS life skills (e.g., meal planning, shopping on a budget, food preparation/cooking skills, and gardening) and sports nutrition education to support sustainable healthy eating and adequate PA among HS soccer players. The WAVE program and follow-up evaluations were approved by the Oregon State University (OSU) Institutional Review Board (#6317).

Participants were screened during the consent process based on inclusion criteria: (1) age 14–19 years; (2) enrolled in HS soccer; (3) living with a parent/caregiver; (4) no medical conditions preventing a normal diet; (5) Internet access during the two-year study; and (6) proficiency in English. The WAVE program included 13 schools, 24 soccer teams, and 620 youth participants (51% female; 51% white, 41% Latino, 41% National School Lunch Program participants). Participants were assigned (non-randomized) to intervention (9 schools,) or comparison (3 schools) groups based on geographical location. Only the intervention group received sports nutrition lessons, newsletters, TBWs, and VW experiential learning. The intervention was delivered to teams during the fall soccer season and summer camps. The WAVE HS sports nutrition curriculum was delivered to teams and coaches by a registered dietitian nutritionist (RDN) trained in sports nutrition and who had played collegiate/professional soccer. Prior to intervention, all lessons were pilot-tested and revised based on input from athletes and sports nutrition experts. Topics covered were as follows: hydration; pre/during/post-exercise fueling; body composition/image; maintaining muscle and staying healthy; and eating well while dining out. Newsletters reinforced lessons and provided recipes/tips to meet sports nutrition needs. Three life skill training sessions were taught via TBWs (~1–1.5 h each) focused on meal planning, shopping on a budget, food preparation/cooking skills, and gardening delivered to teams by the Extension Faculty and community partners. WAVE participant’s data were collected using three questionnaires: (1) a demographic and health history questionnaire; (2) a validated sports nutrition questionnaire [18]; and (3) a food frequency questionnaire [19]. Anthropometric data were measured using a free-standing stadiometer and a calibrated Tanita scale (TM-300-A, Tanita Corpl, Tokyo, Japan), while PA assessment data were collected using a Fitbit zip over a 7-day window. All data were collected at baseline and the end of Year 1 and Year 2. For detailed baseline methods and participants characteristics, see Manore et al. [14]. 

### 2.2. Intervention Evaluation and Research Questions

The intervention evaluation was informed by standard process evaluation procedures [20] and similar nutrition-related intervention evaluations [21,22,23,24]. We addressed the following research questions:

(1) Process evaluation: Were the program interventions delivered with high fidelity? Were programs attended by participants?
(2) Best practices: What were the barriers/challenges and best practices for program implementation?
(3) Youth/coach engagement: Did youth participants and coaches enjoy the educational content, approach used, and intervention activities?
(4) Youth applications: Did youth report sharing and applying the knowledge and skills learned to their daily lives?

### 2.3. Data Collection

**Figure 1:**
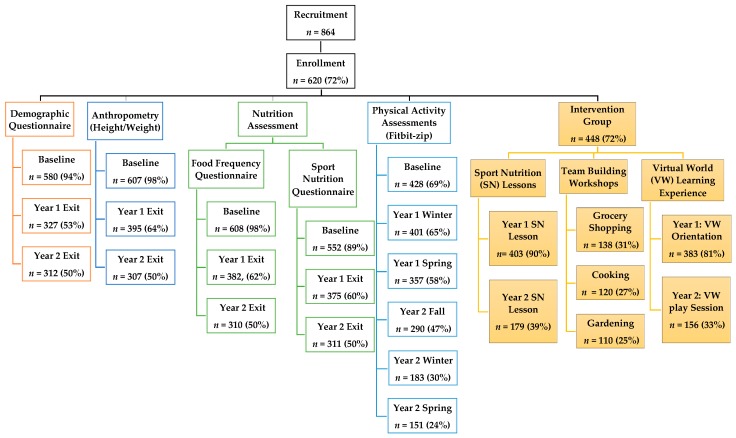
WAVE program youth participant attendance and retention by intervention activities. SN: sports nutrition; VW: virtual world.

Survey and interview questions were reviewed and revised by the principal program investigators (SSW, MMM). The WAVE program research assistant (YM) conducted all interviews. Data were collected from stakeholders at the end of the two-year intervention through qualitative and quantitative measures:

(1) Internal stakeholders (WAVE program coordinator, nutrition instructor, four field team members, and the virtual learning team leader; *n* = 7) were interviewed (30–90 min) through semi-structured face-to-face interviews at work. Each interview was audio-recorded and transcribed by the interviewer. Two independent reviewers (SSW, YM) coded the transcripts and agreed on key themes. Graduate/undergraduate student volunteers (*n* = 27), some of whom were bilingual/bicultural, were surveyed anonymously and asked whether or not they received enough training before implementing the WAVE program in the field, and about protocol adherence among staff.
(2) External stakeholders were WAVE youth participants (*n* = 100) and intervention soccer coaches (*n* = 9). A sign in/out process was used to track youth participation and engagement in each activity, a percentage of engagement was calculated for each activity (Figure 1). Survey links were sent out to intervention youth participants via email and text messages and opened for three weeks with two reminders sending out to youth in between. The youth survey was not anonymous and asked participants to rate each intervention activity based on their expectations, engagement, and enjoyment. Youth received a 5 U.S. dollar gift card as an incentive for participation. Soccer coaches completed a non-anonymous paper survey rating program activities based on their expectations and engagement, and provided rational for their ratings. Coaches also provided their perceptions of changes observed in eating behavior, PA, and life skills among their HS soccer players as a result of WAVE study participation.

### 2.4. Data Analysis

Descriptive statistics (mean/percentages) were calculated from a participation sign-in/out tracking sheet, and for each of the surveys. Staff interview audio recordings were transcribed verbatim and transcriptions were checked against audio-recordings to ensure accuracy. All transcripts were coded by two coders and themes identified. Data were then imported into Nvivo (QSR International Pty Ltd. Version 11, 2017, Doncaster, Australia) for analysis. Coding was performed manually with thematic codes reflecting the questions asked. Within questions, codes were derived as the classification proceeded. Interview response codes were grouped into the following areas: process data on program fidelity, best practices for implementation, youth and coach engagement in intervention activities, and youth application of knowledge and skills to their lives.

## 3. Results

### 3.1. Youth Engagement, Attendance, and Retention

Figure 1 shows youth engagement, attendance, and retention. Overall, 89–98% of youth participants completed baseline demographic, anthropometric, and nutrition assessments. The PA assessment completion rates ranged from 24% to 69%, depending on the time point measured. Year 1 and 2 average participation retention rates were 59% and 45%, respectively. In Year 1, sports nutrition lessons were focused on hydration and exercise fueling, with a 90% attendance rate. In Year 2, the attendance rate was 39%, and the sports nutrition lessons covered body composition/image, maintaining muscle and staying healthy, and eating well while dining out. The key reasons for lower attendance in Year 2 were attributed to seniors graduating and players dropping out of soccer (see discussion for details). Overall, the TBW attendance was 25–31%. This lower attendance rate could be attributed to the TBWs being held on the weekends or after school, and in off-season for soccer.

### 3.2. Program Implementation Challenges, Best Practices and Staff Observations

Staff (*n* = 7) self-rated (range = 1 to 5) their adherence to the study protocol as high (mean = 4.7/5; 94%) and this was confirmed by the graduate/undergraduate student volunteers (*n* = 27) who rated staff’s adherence high (84.6%). Over 90% of WAVE program volunteers thought they had received sufficient training for their position and responsibilities, and that the study was well organized.

*Recruitment and Retention Strategies*. Soccer coaches were recruited through OSU 4-H Soccer (summer program); then soccer youth and parent participants were recruited through the HS soccer program (fall program). For the HS coach recruitment, staff considered a face-to-face presentation by the principle investigator as best practice. The success of youth participant recruitment varied based on the individual coach’s encouragement and his/her relationship with the soccer team. The variability of coach engagement challenged the field team staff to adapt different recruitment strategies based on each coach. Every effort was made to communicate with coaches, youth and parents in a variety of ways (emails, texts, postcards, social media, and/or phone calls); bilingual communications were also provided.

*Enrollment*. The enrollment process included three meetings: (1) an information session, (2) the collection of consent/assent forms, and (3) the collection of a demographic survey. After the recruitment packages were provided to youth and their parents, an in-person meeting was scheduled at the coach–parent soccer meeting. Adequate program staffing to cover enrollment meetings was reported as the biggest challenge. For parental/guardian enrollment, the best practice was to allow either online consent or in-person enrollment. All materials were available in both English and Spanish, and at least three staff at each enrollment meeting were bilingual. 

*Demographic, anthropometric, and nutrition assessments*. At baseline, the average assessments completion rate was high (89–98%), reflecting the organizational skills and engagement from the WAVE program field team staff (Figure 1). Surveys were administered via Qualtrics online/offline using program iPads or participant’s personal computer/phone. The biggest challenge was to follow-up with participants who missed the anthropometric assessments. Other major challenges included being short-staffed during some assessments, long travel times to schools (50–80 min one-way), and the timing of anthropometric measurements prior to soccer practice. For accurate anthropometric data, the protocol required participants to fast for 4 h, avoid MVPA in the previous 12 h, wear light weight clothing, and use the restroom 30 min before the assessment. To assure assessment completion, the best practice was to build a relationship with school office and coaches to improve communicating with youth about follow-up assessments. Food-related incentives were reported as the favorite of the youth participants.

*PA assessments*. As mentioned above, PA assessments had the lowest participation rates (Baseline = 69%, Year 2 = 24%) (Figure 1). Participants were required to wear a Fitbit-zip for 7-day, and complete a daily behavior checklist about sport activities, food intake, sleep, injuries, and bowel health during this period. The criteria set for acceptable PA data was to wear the Fitbit-zip a minimum of 2 days (8 h/d) within a 7-day period. Although 537 (86%) participants picked up their Fitbits at baseline, only 69% had valid data for analysis (2 days of wearing for 8 h). Low completion may have been due to the following: (1) late Fitbit returns, (2) Fitbit malfunction, and/or (3) forgetting to wear the Fitbit. Based on field notes, the average late return rate was 27%, with the highest return rate during soccer season (80%) and lowest return rate during spring term assessment (64%, out of soccer season). During soccer season, staff attended soccer practices and worked with soccer coaches to distribute and collect Fitbits. During the off-season for soccer, the field team reported that the biggest challenge was communicating with participants about Fitbit returns. The best practices reported were as follows: (1) working with school office staff to help collect Fitbits; (2) texting a reminder to youth the evening before Fitbit collection; and (3) giving extra incentives (5 U.S. dollars gift card) for returning the Fitbit on time.

*Sports nutrition lessons*. The same sport dietitian instructor delivered all face-to-face lessons to participants. The major challenges to delivering the lessons during the two-month soccer season were travel time to each school, transportation of teaching materials and equipment to the classroom, and classroom management. The instructor identified four best practices: (1) let participants, and not the instructor, rotate rooms for each activity; (2) use the university campus summer camp venue instead of individual HSs; (3) use PowerPoint clicker questions to keep participants engaged in the classroom lessons; and (4) use an instructor assistant to distribute lesson materials, snacks, and help with classroom management. The nutrition instructor shared this information:

“…they [participants] learned a lot of things they didn’t know. Some girls told me that they have made [sport] bars [No Bake Energy Bars] for half-time, fueling during their sports. They are drinking more water and trying to keep hydrated. I had one participant text me about how she used one of the recipes we did at the TBW, when her parents were gone and she needed to cook for herself. People were actually using what we were teaching them.”

*Team-Building Workshops (TBWs)*. All TBWs were face-to-face group lessons with hands-on activities and team competitions. Each workshop (1–1.5 h) had different learning objectives and facility requirements, but all had similar challenges identified by field staff: (1) difficulty in scheduling youth during the off-soccer season; and (2) less participant engagement when not in-soccer season. The field staff indicated that when coaches required or encouraged attendance participants were more engaged, especially males and Latino youth. For TBW participation, best practices included the following: (1) coach encouragement and school coordination; (2) incentives; (3) creating fun and competitive activities that provided hands-on experiences; and (4) providing transportation from school to event location. The TBW staff shared the following:

“The students learned a lot about gardening by hands-on involvement and in a structured educational workshop environment. … The students were put into groups and asked to create a recipe from the food they harvested that day. It helped the students to learn how to develop a recipe from the food that is available to them and cook from scratch versus preparing or eating processed foods. [The] Life skills learned was to cook for themselves. … I heard them say, “I’ve never been in the farm before”; “this is fun to use these tools”. They don’t mind to be muddy…The students bonded with one another and their coaches. They had a great time, and there was a lot of laughter and working well together.”

*The virtual world (VW) experiential learning environment*. The biggest challenges to the VW were having access to stable and strong internet connectivity at each school, enough laptops for each participant, keeping a low participant-to-instructor ratio, and adequate time to do the activity. Time for face-to-face orientation is critical for youth to learn about the VW and log in themselves. The WAVE technical team used written scripts to keep instruction consistent with groups, and observers kept notes for future improvements.

### 3.3. Youth Enjoyment

**Figure 2:**
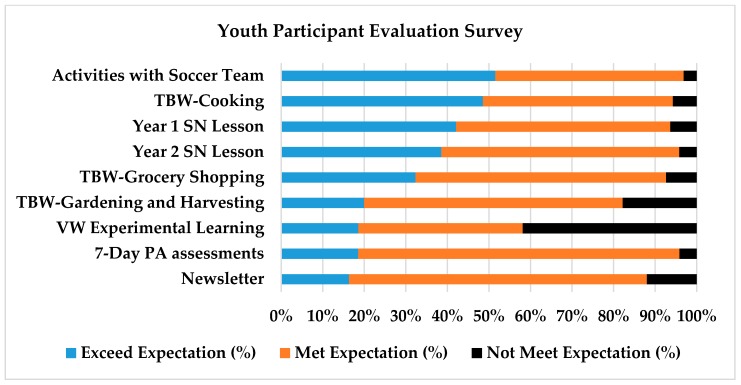
Youth rating on intervention activities. TBW: team-building workshop; SN: sports nutrition; VW: virtual world; PA: physical activity.

To determine youth enjoyment, participants rated each WAVE intervention activity based on how well each activity met their expectations (exceeded, met, not met) (see Figure 2). The top three activities that exceeded youth expectations (>90%) included the following: (1) participating in activities with their soccer team; (2) the cooking TBW; and (3) sports nutrition lessons. The following statements represented the reasons behind their choices:

“My favorite part of the WAVE project was spending more time with my soccer team to learn how to better nourish myself.”

“Extra team bonding that was a lot different compared to practice or team dinners.”

“Being with my teammates and learning what was good for our health in a fun way.”

“I really enjoyed the hands on activities like cooking with my team.”

“I enjoyed learning about sport nutrition and the helpful tips regarding what to eat before workouts and games.”

Youth participants rated three additional WAVE intervention activities as follows: (1) the WAVE newsletter (87%); (2) the gardening TBW (82%); and (3) the VW experiential learning environment (59%). The WAVE newsletter was rated highly, with 61% of participants saying they had shared the newsletter with their families and friends. Youth attending the gardening and harvesting TBW activity (25%) rated it highly, with 60% reporting the educational content was appropriate and that they applied what they learned to their daily life. The VW experiential learning environment was rated lower than other activity, which was most likely due to the program still being in the development phase.

Comments about the VW included:

“The part of the WAVE project that I liked the least was Rippleville [name of WAVE VW] because I didn’t understand it all that much and every time I tried to use it, it didn’t really work out that great, I thought it was a great idea, …, I never got to use it that much…. except…with the staff.”

**Figure 3:**
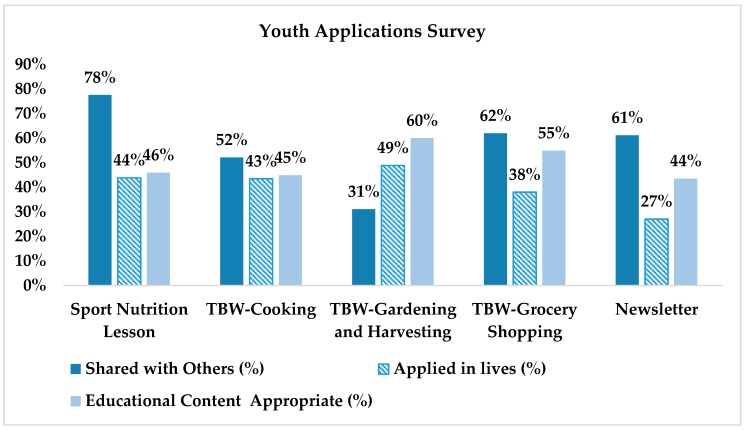
Youth rating of the WAVE educational components and learning outcomes. TBW: team-building workshop.

Youth were also asked if they had applied what they learned from the WAVE program to their lives or shared with others. Youth participants shared what they learned from the sports nutrition lessons (78%), the grocery shopping TBW (62%), and newsletters (61%) with their families and friends (Figure 3). Youth reported they were most likely to apply the knowledge and skills they learned from sports nutrition lessons (44%), the gardening TBW (49%), and the cooking TBW (43%). Finally, the educational content from the grocery shopping TBW (55%) and the gardening TBW (60%) was rated higher than the other activities. Overall, the sports nutrition lesson had the highest percentage of participants (18%) providing scores with respect to a combination of education content and learning outcomes.

### 3.4. Coach Engagement

**Figure 4:**
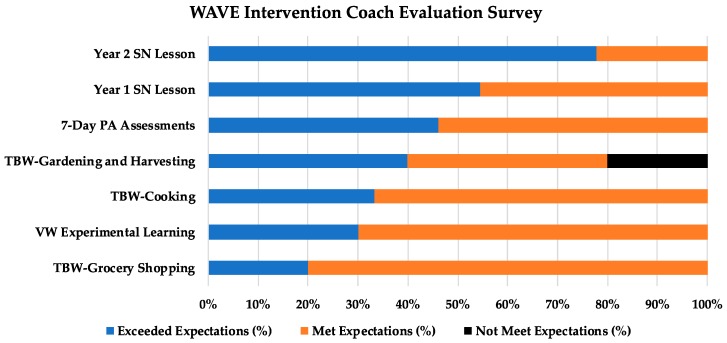
Coach rating of WAVE intervention activities. TBW: team-building workshop; SN: sports nutrition; PA: physical activity; VW: virtual world.

Figure 4 shows how the intervention coaches rated the WAVE program activities. Overall, the coaches agreed with participants that the sports nutrition lessons exceeded their expectations, especially the lessons on body composition and image (Year 2: 70%) and hydration (Year 1: 55%). For the coaches surveyed (*n* = 9, 45% of intervention coaches), all attended the sports nutrition lessons with their players. Coaches also reported that youth participants discussed nutrition and exercise fueling after the sports nutrition lessons. Less than 50% of the surveyed coaches attended the TBWs, which typically occurred at the weekend/after school, thus rating them lower. Only one coach rated the gardening and harvesting TBWs as below their expectations, which was due to poor weather and mechanical problems with their bus, which delayed the students. The coaches shared the following observations about their participation of their athletes regarding sports nutrition and dietary knowledge/behavior:

“More talking and discussions about nutrition and what food were consumed during seasons”

“Players have been more responsible with their diet, especially during the season. They have done a much better job avoiding fast/junk food than in previous years and are better educated.”

“Players were definitely more aware of hydration and nutrition needs and were able to help other players understand the importance.”

“I have seen an increase in the awareness of what is going into their body…. Most of the players now eat a half-time snack and make sure that they are properly fueled before kickoff. Thank you for the time you’ve spent educating them.”

## 4. Discussion

This is the first study to assess the feasibility, best practices, youth engagement/enjoyment and application, and pitfalls of implementing a two-year integrated (research, education, and extension) PAN-FCS life skills intervention among HS athletes. Internal and external stakeholders were surveyed and/or interviewed for information on process and impact of the program. Results showed that a two-year educational and experiential learning intervention is feasible for HS athletes; however, there were a number of hurdles to overcome for success to occur. These hurdles included recruitment and retention, training of staff and volunteers, coach involvement, and keeping youth engagement high. Each of these areas is discussed below. Results also showed that athletes enjoyed learning about sports nutrition, importance of PA, and life skills that could immediately be applied to their lives as an athlete. 

### 4.1. Recruitment and Retention

Overall baseline recruitment and assessment completion rates were high (>70%). Our recruitment strategy relied on referrals from a trusted source and the relationship between the coach/player. When a strong coach/player relationship was present, player participation levels were high and consistent. When coach engagement was lacking, more program staff outreach was required to maintain participation. Program continuity was maintained between staff/volunteers and participants by using the same staff and/or bilingual/bicultural volunteers to follow-up with each school. In Year 1 (baseline), the sports nutrition lesson attendance rate (90%) and assessment survey response rates (89–98%) were higher than reported by others (79–89%) implementing obesity prevention intervention studies (baseline data) among HS students [25,26,27]. These programs included non-athletes and lasted less than 3 months. During Year 2, our retention rate decreased to 39%. Cui et al. reviewed 43 minority youth (aged 11–18 years) obesity prevention and treatment trials lasting less than 1 year [25]. They found the average retention rate to be 81%. To date, no study has completed a nutrition, PA, and life skills obesity prevention intervention lasting longer than 3 months in HS athletes. Ranby et al. completed a 3-month intervention in female HS athletes (*n* = 807), with a 9-month follow-up focused on reducing substance abuse and improving healthy weight loss behaviors. At 3 months their retention rate was 75%, and at 9-months the post-intervention retention rate was 50% [28]. A weight management intervention by Daly et al. (2016) showed that Latina youth were more likely to drop out due to social barriers [29]. We found no ethnicity difference between drop-outs (40%) and completers (45%) at the end of our two-year intervention. In Year 2, two key factors contributed to our lower retention rate: (1) seniors graduating from HS at the end of Year 1, with these initial participants not being available for Year 2; and (2) Participants no longer playing soccer in Year 2.

### 4.2. Training Staff and Volunteers

Overall, staff (94%) and trained volunteers (84.6%) rated the study as having high implementation fidelity, meaning the program implementation accountability was high and the study protocol was feasible. Each phase of program implementation had different challenges, but staff were able to accomplish the tasks using best practices. Staff rated coach involvement as critical to program implementation adherence.

### 4.3. Coach Engagement

Coach engagement was high; all surveyed coaches (*n* = 9) attended the sports nutrition lessons and rated them highest among all WAVE activities. Thus, coaches were learning sports nutrition information and implementation skills along with their athletes (5–6 h of sports nutrition education). We also provided coaches with handouts and laminated posters for the locker room to remind students of the sports nutrition messages athletes had learned. Our coaches observed players having more nutrition awareness, and conversations around hydration and exercise fueling. Having the coaches recognize the value of sports nutrition for their athletes is imperative to support educational programs such as WAVE. Few HS coaches have accurate sports nutrition knowledge to properly make nutrition recommendations for their athletes [30,31,32]. We did not assess the sports nutrition knowledge of our coaches; however, Jacob et al. reported low (70–73%) sports nutrition knowledge in their HS coaches (*n* = 41) [33]. They found that when coaches completed two 90-min sessions on sports nutrition over a 2-month period they significantly increased nutrition knowledge to 82%. Thus, even limited sports nutrition training for coaches can significantly improve the messages they provide to their athletes. Bleski et al. also found that community-level, volunteer HS coaches attending a sports nutrition session could significantly increase their sports nutrition knowledge on use of sports drinks (86% to 99%) and exercise fueling (64% to 81%), and improve self-efficacy in understanding young athletes’ nutritional needs (6.8 to 9.0) [34].

### 4.4. Youth Engagement

Youth participants reported that ‘activities with their soccer team’ represented the most enjoyable component of the WAVE program. The feedback from youth indicated that they liked having hands-on activities and competitions with their teammates that occurred outside of soccer practice. Overall, 78% of participants shared their sports nutrition lessons with their friends and 44% reported applying it to their lives. Research shows the importance of peer influence on behavior change among adolescents [35] and the strong influence peers have on food choices [36,37]. Eisenburger and Neumark (2010) examined the eating behaviors of adolescents (*n* = 2526; 32% HS students) over a five-year period. If female participants reported friends dieting at baseline, they were more likely to report chronic dieting, unhealthy and extreme weight control behaviors, and binge eating at the end of the five-year period. If male participants had friends dieting at baseline, they were more likely to report extreme weight control behaviors at the end of 5 years [38]. The impact of team membership on eating and food selection may be even stronger since athletes spend so much time together. Smart and Bisogni examined factors influencing food choices in male hockey players (*n* = 25). They found that athletes reported spending most of their social time with team members, and that these teammates influenced their food choices and eating practices [36]. Based on these data, it is clear that peers influence each other’s eating behaviors and food choices. Thus, sport team interventions provide a unique environment to enhance positive behavior changes associated with food and beverage selection, making them a practical setting for health promotion among adolescents.

### 4.5. Strengths and Limitations

This study had several strengths. First, a two-year pilot study preceded the WAVE two-year intervention to ensure all material and information presented were research based and addressed HS soccer athletes’ sports nutrition concerns. The pilot study also helped assure that activities and lessons were enjoyable and applicable. Second, this is the first study to demonstrate the feasibility, fidelity, and best practices of implementing a two-year obesity prevention program among HS athletes using input from internal and external stakeholders. Another indication of feasibility was the willingness of HS soccer coaches to participate, recruit their teams, and support the WAVE intervention activities. Third, our qualitative feedback from coaches and youth support the positive diet and life skills behavior changes we found based on qualitative data [15]. Finally, the WAVE program reached a diverse group of youth soccer players, including Latino (41%), low income (41%, based on National School Lunch Program participation), and male youth (49%).

This study also had limitations. First, the retention rate declined over the two-year intervention due to participants graduating or dropping out of soccer. We recruited soccer teams for the WAVE intervention, which meant that some participants were in their last year of HS in Year 1. In addition, in Year 2, some team members dropped out of soccer and were no longer on the team. One approach to reducing participant dropout is to offer the program in one year and only during the soccer season. Second, the TBW attendance was low compared to other WAVE activities, which highlights the need to anticipate attendance barriers and develop creative solution when engaging youth throughout the school year, including out-of-soccer season and off campus. In addition, youth have numerous activities and jobs that may have conflicted with attendance at the TBW. Third, the VW learning environment, which was designed to reinforce the sports nutrition lessons and provide experimental learning, was not fully developed when the intervention began. Thus, the potential of this educational tool could not be adequately tested. Based on preliminary data from WAVE program focus groups, we found that VW experimental learning remains promising as a way to reinforce nutrition messages and provide a method of practicing new skills in the context of a game. This educational tool should be explored further.

## 5. Conclusions

The process, best practices, and youth engagement evaluations of the two-year WAVE obesity prevention intervention had two significant outcomes. First, based on our high coach satisfaction and youth engagement, it is feasible to use sport teams/clubs to engage youth and young adults. The sport team/club environment represents a unique setting for obesity prevention programming, and offers potential for helping youth acquire the knowledge and skills to increase positive heath behaviors related to healthy eating, adequate PA, and life skills (e.g., meal planning, shopping, cooking and gardening). Youth wanted to learn how to eat to improve their sport performance; thus, youth were interested in participating. Given the high prevalence of obesity in adolescents, especially in youth aged 12–19 years, this is a promising public health approach to obesity prevention. Second, we report best practices for implementing an obesity prevention program in HS athletes, based on internal and external stakeholder data. Overall, implementing the best practices identified in the WAVE intervention will help assure the success of direct future programing in this area.
